# Toward Energy-Independence and Net-Zero: The Inevitability of Subsurface Storage in Europe

**DOI:** 10.1021/acsenergylett.2c01303

**Published:** 2022-07-08

**Authors:** Johannes M. Miocic, Juan Alcalde, Niklas Heinemann, Ignacio Marzan, Suzanne Hangx

**Affiliations:** † Energy and Sustainability Research Institute Groningen (ESRIG), University of Groningen, 9747 AG Groningen, The Netherlands; ‡ Geosciences Barcelona, GEO3BCN-CSIC, 08028 Barcelona, Spain; § School of Geosciences, University of Edinburgh, Edinburgh EH8 9XP, United Kingdom; ∥ Spanish Geological Survey, IGME-CSIC, 28003 Madrid, Spain; ⊥ Department of Earth Sciences, Utrecht University, 3584 CB Utrecht, The Netherlands

The rise in gas prices, accelerated by the war in Ukraine, has exposed the volatility of the energy market and Europe’s need to achieve independence from external supplies from politically sensitive areas. Reaching such independence, while simultaneously complying with climate agreements, will inevitably require a substantial expansion of subsurface energy and byproducts storage options.

Europe is a net importer of oil and gas, making its energy market sensitive to external disruptions of supply and demand.^1^ This dependency on external energy sources has led to current and past supply insecurity, resulting in increased energy prices for household consumers and industry. As a result, Europe is currently experiencing levels of inflation not seen in over 30 years, largely driven by the increase in oil and gas prices.^2^

Although the early 2022 energy price hike can be largely attributed to the current political instability, a surge in supply and demand problems had already put pressure on the global energy market in 2021. On the European supply side, long-term effects such as decreasing gas production from the well-developed North Sea plays a role, and the planned closure of the Dutch Groningen gas field have made Europe more reliant on oil and gas imports.

Storage of natural gas is used to balance the supply and demand in the energy system, allowing the buffering of price hikes by supplying gas stored during times of low demand (e.g., summer) in times when demand exceeds production (e.g., winter). In 2021, European gas storage sites were substantially depleted after an unusually cold winter. The high gas demand to refill these storage sites during spring and summer of 2021,^3^ and the worldwide increase in energy demand after most countries lifted COVID restrictions, prevented gas storage sites from being sufficiently recharged. Additionally, unpredicted short-term disruptions, such as maintenance work on European gas fields, storms in the Gulf of Mexico, and interruptions at major Russian processing facilities, resulted in a global increase in demand for seaborne liquified natural gas (LNG). Thus, the following winter season started at a low base level of gas reserves, and Europe was severely dependent on gas imports piped from Russia.

Large-scale storage of energy is an effective way to increase energy security and reduce the reliance on short- and medium-term disruptions of energy imports. As an example, many countries hold strategic petroleum reserves as an emergency backup, a lesson learned from previous politically motivated oil embargoes. However, while Europe became increasingly dependent on gas imports, gas storage capacity has not been expanded in recent years. Realizing the need to reduce its energy vulnerability, Europe currently seeks to diversify its gas supply by increasing the LNG supply share.^4,5^ At the same time, coal is planned to be phased out in favor of natural gas to curb CO_2_ emissions as stated in climate agreements.^6^ If these increased gas volumes cannot be generated within Europe, an even greater need for net imports should be accompanied by an increase of the European gas storage capacity.

In the longer term, an increased deployment of intermittent renewable energy sources (wind, solar) will require additional energy storage, likely in the form of hydrogen. Finally, to comply with net-zero targets, permanent CO_2_ storage is needed and will lead to an even greater reliance on storage activities. Therefore, we argue that the development of more geological storage in both the short- and long-term is inevitable, and storage site assessment and detailed asset planning on an international scale is crucial and needs to commence today.

## 1 Europe’s Current Subsurface Energy Storage Capabilities

European emergency stocks of crude oil and petroleum products are able to cover at least 61 days of consumption, or 112 million tonnes of oil, as is obligatory for EU member states.^7^ In addition, 55 million tonnes of oil are commercially stored, predominantly in salt caverns.

**Figure 1:**
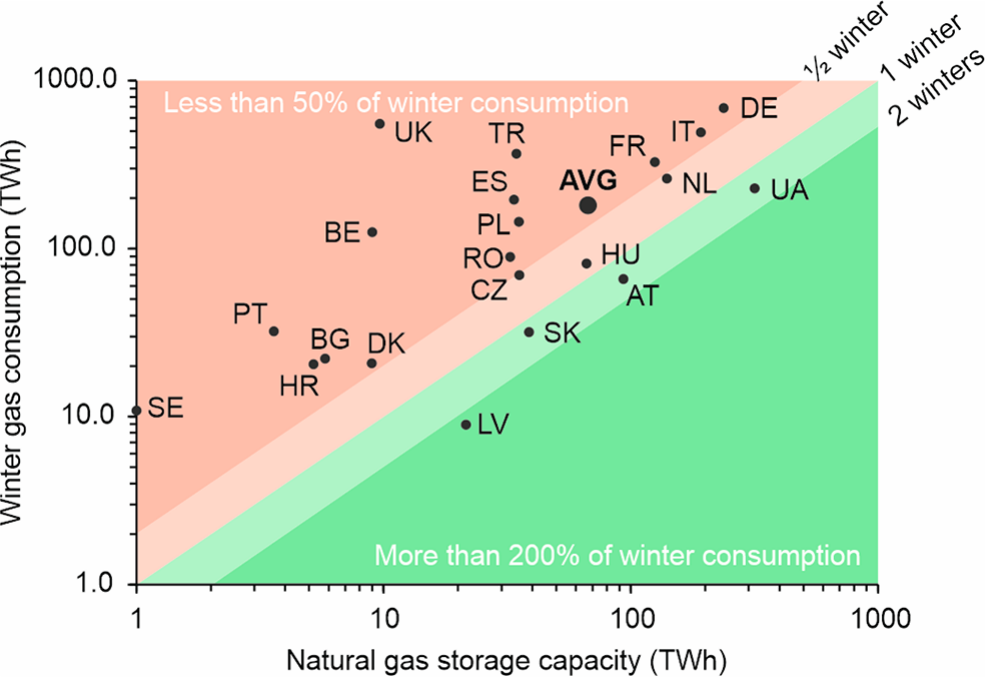
Subsurface natural gas storage capacity (AGSI+^3^) of European countries to meet their winter energy consumption (October 2020-March 2021; Eurostat:^9^ UK’s winter 2020 gas consumption from AGSI+^3^). Country codes according to ISO 3166-1 alpha-2 standard (https://www.iso.org/iso-3166-country-codes.html). AVG indicates the average for the European countries depicted. European countries not shown do not have any gas storage facilities.

By contrast, natural gas is only stored in commercial facilities, such as surface tanks, depleted oil and gas fields, salt caverns, and saline aquifers.^8^ Overall, 1484 TWh of working gas are available across 174 underground storage sites, distributed over 20 European countries (excluding Russia). Additionally, 18 commercial storage sites are planned or under construction, potentially increasing the gas storage capacity by 164 TWh (+11%). At its current gas storage capacity, Europe can store 43% of its total winter gas consumption (based on 2021 demand; see Figure 1). Only Austria, Latvia, Slovakia, and Ukraine have higher storage capacities than their winter demand. Furthermore, 10 countries do not have any storage facilities, thereby solely relying on imports to supply the 141 TWh worth of combined natural gas that they consumed in winter 2021. An EU-wide natural gas storage policy is currently under discussion, focusing on designating storage facilities as critical infrastructure and filling targets for winter season buffers,^5^ ensuring a lower need for imports during the heating season. Despite plans for expanding the storage capabilities across Europe, the current lack of storage highlights Europe’s vulnerability to fluctuations in gas supply, and thus price volatility.

## 2 The Future Subsurface Storage Landscape

### 2.1 Meeting Current Natural Gas Demands: Increased Gas Storage

Natural gas is seen as a key fuel for heating and electricity generation during the transition toward a renewable energy system.^10^ In light of this, Germany would need an additional gas power capacity of 20–30 GW to replace coal, equivalent to doubling its gas power plants currently in operation.^11^ To reduce interdependencies and improve negotiation capacity for gas supply, Europe is promoting a diversification of its suppliers by switching from piped natural gas to shipped LNG^5^ from the US, Qatar, and/or Africa.^12^ Although LNG trade is flexible, its supply is logistically complex, requiring regasification infrastructure at the entry ports and capacity to handle a fluctuating gas supply. Therefore, a strong local or global increase in LNG demands, or the occurrence of eventual supply chain problems, such as the 2021 Suez Canal blockade or the more recent COVID related lockdown in Shanghai, leads to price volatility.

Only when coupled with an expansion of gas storage facilities could this diversification strategy make Europe more resilient to short-term disruptions of gas supply, (inter)national conflicts, or natural disasters. Natural gas storage in times of low demand and price would allow for temporary stabilization of internal supply and price. The higher the predicted volatility, the higher the storage capacity needs to be.^13^ Given current political developments, as well as the urgency to remain focused on meeting climate agreements for reducing CO_2_ emissions, the time for the development of a pan-European gas storage program is now!

### 2.2 Transitioning to Renewable Energy Forms: Seasonal Energy Storage

Although natural gas is predicted to play a crucial role in the energy transition, Europe’s long-term goal to achieve the energy transition^6^ and meet zero-emission targets^14^ requires increasing energy efficiency^15^ and shifting to renewable (wind, solar) and low-carbon (nuclear, geothermal) energy sources. The intermittent nature of renewable energy sources makes the implementation of large-scale (seasonal) energy storage necessary to compensate for periods of low energy production. Given the vast amounts of renewable energy that will need to be stored (TWh-range), the subsurface is the only candidate that can provide the required storage volumes. The conversion of excess renewable electricity to hydrogen and its seasonal storage is widely identified as the most promising solution to optimize the renewable energy system and simultaneously decarbonize large-scale energy storage,^16^ with compressed air energy storage^17^ and underground thermal energy storage^18^ also being appraised. Additionally, replacing conventional fuels with synthetic ones, such as synthetic methane or kerosene, will help to lower carbon emissions from sectors that are otherwise difficult to decarbonize (e.g., domestic heating, aviation^19^). However, these synthetic fuels will also need storage.

Each type of energy storage requires specific subsurface characteristics, and available options are not equally distributed across Europe. The North Sea offers substantial storage capacity in depleted and soon to be depleted gas fields that could meet the energy storage requirements of several countries, while renewable energy production is more decentralized and often does not occur in the vicinity of suitable storage locations. Additional factors, such as social acceptance, will crucially influence storage site availability, as multiple subsurface storage projects have been abandoned in Europe in the past due to lack of social support.^20^ This, in turn, highlights the need for transnational collaboration between countries with different energy characteristics and needs (i.e., those with suitable storage locations and those with renewable energy surplus). This strategy should help in designing a subsurface landscape that optimizes the European storage capacity.

### 2.3 Mitigating Climate Change: Permanent CO_2_ Storage

In parallel to Europe’s energy needs are the climate targets aimed at drastically reducing carbon emissions. The transition to a clean energy system encompasses increasing energy efficiency and switching to cleaner fossil fuels (natural gas) and renewable energy. In this low-carbon context, large-scale geological storage of CO_2_ is unavoidable to mitigate residual emissions and to meet emission reduction targets.^21^ The European Commission calculates that at least 5 Mt of CO_2_ should be stored annually by 2030,^22^ with other studies suggesting this may increase to 350 Mt CO_2_/yr from 2030 onward.^23^

Carbon Capture and Storage (CCS) is currently the one option to decarbonize combustion power stations and CO_2_-intensive industries, such as cement and steel production. Additionally, Bioenergy with Carbon Capture and Storage (BECCS) is predicted to be vital to meet the 2050 net zero targets,^24^ also requiring large subsurface storage of CO_2_. It should be noted that geological CO_2_ storage in the deep subsurface entails the permanent storage of CO_2_, meaning that CO_2_ storage sites cannot be repurposed for other storage uses. This again strengthens the call for adequate action now to design a subsurface landscape in which all these technical solutions can find their rightful place.

## 3 Call to Action: Designing the Subsurface Landscape

In conclusion, if Europe wants to decrease its reliance on energy imports, while also meeting its carbon emissions targets, large-scale subsurface storage is inevitable, and the current storage capacity needs to be increased. Developing more storage will not help to resolve the current gas crisis but will make Europe more resilient by increasing energy security and reducing CO_2_ emissions in the future.

To facilitate energy security in the coming years, a European strategic gas reserve should be encouraged in addition to the expansion of commercial gas storage in Europe. Alternative ideas, such as the declaration of accessible cushion gas as strategic reserves in commercial gas storage sites to lower investment costs and increase energy security, should be discussed. For the future development of subsurface storage, Europe-wide site selection will be decisive. However, with the envisioned coexistence of subsurface storage activities, in addition to energy production strategies, such as natural gas and geothermal energy, an overarching subsurface utilization strategy is needed. Many current natural gas storage sites are located in salt caverns, which are also preferred locations considered for hydrogen storage due to the tightness and limited reactivity of the structure. However, their storage volumes are substantially smaller than those of sedimentary structures. Utilizing oil and gas fields increases storage capacity faster, and many North Sea fields are approaching their end of life in the next decade, highlighting that increasing storage capacity needs to be placed against the time frame in which storage space becomes available. While there is an apparent abundance of storage locations, linking storage type and location to energy and CO_2_ producers, energy consumers, existing infrastructure, and social acceptance must be part of an optimization strategy to guarantee efficient storage. It is urgent to develop workflows to determine the suitability of storage assets for the different storage technologies to optimize Europe’s subsurface landscape. Hence, scientific investigations, transnational strategies, and an integration of current storage facilities with future ones, are needed *now*.
